# Daily physical activity, coffee and energy drink consumption, and sleep patterns among Chinese elite professional esports athletes: a case study of Zhejiang Regans Gaming

**DOI:** 10.3389/fpubh.2025.1557533

**Published:** 2025-02-26

**Authors:** Zhihui Pang, Lide Su, Yang Zhang

**Affiliations:** ^1^School of Humanities, Inner Mongolia University of Technology, Hohhot, China; ^2^College of Physical Education, Hunan Normal University, Changsha, China; ^3^Independent Researcher, Windermere, FL, United States

**Keywords:** caffeine, exercise, gaming, health promotion, insomnia, nutrition

## Abstract

**Purpose:**

Esports is increasingly recognized as both a legitimate sport and profession. However, evidence on the health behaviors of esports athletes remains limited. Due to the nature of their activity, these athletes face occupational health risks associated with prolonged sedentary behavior, sleep deprivation, and excessive energy drink consumption. This study aimed to document the health behaviors of professional esports athletes.

**Methods:**

This case study sampled athletes from Zhejiang Regans Gaming, who are full-time professionals competing at the elite level. Data were collected over three training days, with moderate-to-vigorous physical activity and sleep patterns monitored using the Apple Watch Series 9.

**Results:**

Athletes trained for an average of 13 h daily in a seated position, engaging in only 35.6 ± 0.9 min of moderate-to-vigorous physical activity. The extended training hours led to delayed sleep schedules, with a typical bedtime of 3:05 a.m. Sleep quality was poor, with athletes averaging 45.2 ± 13.0 min awake in bed, 392.8 ± 13.9 min of total sleep, and a sleep efficiency of 89.7 ± 3.0%. Likely due to sleep deprivation, athletes consumed an average of 2 cups of coffee and 2.5 ± 0.4 bottles of energy drinks daily.

**Conclusion:**

This study offers firsthand evidence of the concerning health behaviors among Chinese elite professional esports athletes. Physical activity levels and sleep quality are notably poor, while energy drink consumption is prevalent. Team managers should monitor sleep quality and energy drink consumption, while governing bodies must recognize the long-term risks of excessive sedentary behavior in esports.

## 1 Introduction

In 2024, the International Olympic Committee officially recognized esports as a category of Olympic-style competitions, representing a significant shift from its origins as gaming entertainment to its establishment as a formal competitive sport. In China, esports athletes are officially recognized as a profession by the Ministry of Human Resources and Social Security, with various gaming titles being introduced and organized for professional competitions. As the industry expands roughly in its initial stage, it is imperative to prioritize the health and well-being of esports workers, particularly professional athletes, in tandem with its overall development. Lessons must be learned from past tragedies, such as the deaths of athletes during the Baiyin ultramarathon — a major historical disaster attributed to inadequate safety measures by event organizers and gross negligence by Chinese mainstream academics (1) during the rapid expansion of mass marathons in China. Considering the incomplete laws and regulations in China’s esports industry, providing direct evidence of athletes’ health behaviors could help reverse-engineer a more orderly industrial development.

The unique demands of esports create suboptimal working conditions for both professional athletes and underage trainees, often characterized by prolonged sitting and insufficient sleep as a consequence. Lee and colleagues examined sleep patterns in professional esports athletes specializing in first-person shooter games from South Korea, Australia, and the United States (2). Their findings revealed that professional South Korean esports athletes trained for an average of 13.4 h per day, with many exhibiting depressive symptoms. The study also found that all participants experienced delayed sleep patterns, with a median sleep onset time of 3:43 a.m. and a median wake-up time of 11:24 a.m. Similar sleep disturbances have been reported among Brazilian esports athletes (3) and Norwegian amateur gamers (4), suggesting that prolonged sitting, poor sleep quality and daytime fatigue may be prevalent across esports athletes globally (5). A recent cohort study found that sedentary behavior exceeding 10.6 h per day increased cardiovascular mortality risk, even among physically active individuals (6). Consequently, excessive esports training may contribute to early mortality. Moreover, professional esports athletes often experience occupational injuries linked to prolonged sitting, with musculoskeletal pain being the most common (7). Poor spinal posture stability (8) from extended sitting contributes to frequent neck, back, and lumbar pain (9). In China, where the professional community continues to expand, the long-term implications of intensive gaming present a growing public health concern. This is especially critical for the physical health and development of young participants.

The health risks associated with sleep deprivation among esports athletes should not be underestimated. Poor sleep quality is a significant risk factor for ischemic stroke (10, 11) and is a leading cause of premature stroke-related sudden deaths among young individuals in China (12). Additionally, both acute and chronic sleep deprivation have been shown to impair left ventricular function and cardiac re-polarization in healthy young individuals (13). Early cardiac re-polarization, in particular, is a critical factor that can precipitate sudden cardiac arrest (14). Although no cases of sudden death have been reported among Chinese professional esports athletes, several sleep deprivation-related cardiac deaths have occurred among esports industry workers (15). Despite these concerning incidents, the esports industry and academic community have yet to address this significant health risk (16).

Due to prolonged exposure to competitive pressure, sleep deprivation, and advertising influences, professional esports athletes may rely on energy drinks to combat fatigue and enhance focus during competitions. However, what is less widely recognized is that excessive long-term consumption of energy drinks can lead to serious health issues, including arrhythmia, myocardial infarction, and prolonged QT intervals (17). For professional esports athletes, energy drinks may not only obscure the warning signs of physical fatigue but also acutely raise systolic blood pressure and cause re-polarization abnormalities (18), which can potentially trigger sudden cardiac events. This poses a distinct exogenous risk, particularly for professional esports athletes. To the best of our knowledge, no studies — domestic or international — have yet investigated this critical aspect.

Limited research has examined the sleep patterns of esports athletes, primarily using research-grade devices (2). However, advancements in consumer wearable technology now enable accurate, cost-effective monitoring of daily physical activity and sleep patterns. These devices offer new possibilities for research data collection while also allowing athletes to monitor and manage their own health behaviors more effectively. This study investigated the daily physical activity, coffee and energy drink consumption, and sleep patterns of professional esports athletes. Using a case study approach, it aims to raise awareness within the Chinese professional esports community about athletes’ health behaviors and associated risks. The ultimate goal is to promote the synchronized health development of both individuals and the industry.

## 2 Methods

### 2.1 Participants

Zhejiang Regans Gaming (RSG) is a professional esports team based in Pinghu City, Zhejiang Province. The participants in this study were athletes from RSG competing in the Peacekeeper Elite League. The team has won several domestic titles and is one of four teams representing China in the PUBG Mobile Global Championship 2024, where they competed against 45 elite international teams. One of the RSG athletes represented China and secured the gold medal in the Peacekeeper Elite event at the 2022 Asian Games. The entire active roster of the team (*n* = 5) voluntarily participated in this study, each with more than six years of experience as both trainees and professional esports athletes. The exclusion criterion was the failure to provide the required health behavior assessment. The age and body mass index of the athletes were 20.8 ± 0.8 years old and 21.9 ± 1.0 kg/m^2^, respectively. The study was approved by the Ethics Committee of Hunan Normal University (approval number: 2024–821), and all participants provided written informed consent.

### 2.2 Study design and measurements

The study design is a descriptive analysis of athletes’ daily physical activity, drink consumption, and sleep patterns during typical training days. One researcher accompanied the team, providing on-site data collection and analysis. Participants were instructed to maintain their regular daily activities, and researcher interaction with athletes was minimized outside of necessary data collection. Data on three days of targeted metrics were collected in December 2024.

To measure daily physical activity, the original plan was to use an accelerometer-based device to record three to seven days of activities. However, this was deemed unnecessary after the team shared their typical training day schedule with the research team, as detailed in the next section. To measure the time spent in moderate-to-vigorous physical activity (MVPA) and the exercise heart rate response, the Apple Watch Series 9 (Apple Inc., USA) was used. Of note, MVPA is defined in various ways. The World Health Organization, for example, defines MVPA as physical activity performed at >3 METs, or, on a scale relative to an individual’s capacity, as a 5 or above on a scale of 0–10 (19). This study followed the American Heart Association’s practice (also known as Physical Activity Guidelines for Americans) for assessing heart rate responses during physical activity. During moderate-intensity activities, the target heart rate is 50–70% of the age-predicted maximum heart rate, while for vigorous-intensity activities, it increases to 70–85% of the age-predicted maximum. For our young participants, physical activities that elicited exercise heart rates between 100 to 170 beats per minute were considered MVPA. These data were recorded by the Apple Watch and downloaded to a computer for minute-level analysis.

Daily coffee and energy drink consumption was recorded by an on-site researcher. An energy drink is defined as a beverage containing neuro-stimulant ingredients such as caffeine, vitamins (e.g., B_12_), amino acids (e.g., taurine), and herbal extracts (e.g., ginseng). All coffee and energy drinks were provided by the team, and the researcher did not influence the participants’ consumption patterns. Meanwhile, this method enhances the validity of drink consumption data compared to self-reported logs.

At night, participants were asked to wear the Apple Watch on their dominant wrist during sleep and to complete a sleep log documenting their time to bed and time of waking up. All watches were tested for proper functionality before being distributed to the athletes. The validity of the Apple Watch in measuring sleep metrics has been confirmed in previous studies (20, 21), and the latest versions, including the Series 9, are designed to provide even more accurate algorithms for this purpose. Sleep data were downloaded from the Apple Watch the following morning by the researcher for further analysis. It is worth noting that the Apple Watch tracks rapid eye movement, core sleep, and deep sleep, which were summed to calculate total sleep time for this study. The sleep log showed the total time in bed, from which the sleep efficiency — the ratio of total sleep time to total time in bed — was calculated.

### 2.3 Statistics

Data on daily schedule, MVPA, sleep metrics, and drink consumption were organized in an Excel sheet. The data were then processed using R version 4.4.2 (Pile of Leaves) and presented as means ± standard deviations, unless otherwise specified.

## 3 Results

**Figure 1:**
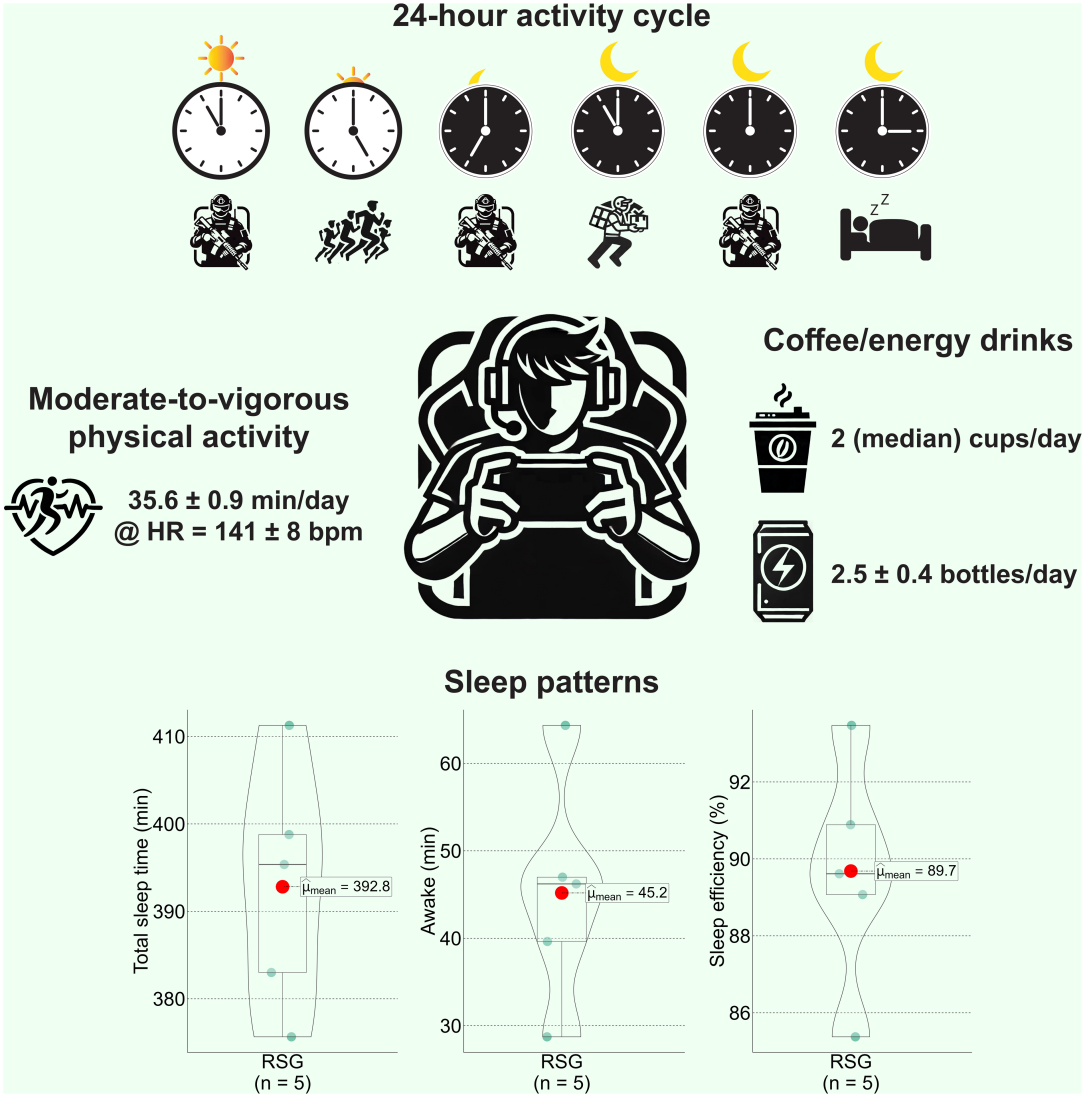
Daily physical activity, coffee and energy drink consumption, and sleep patterns of RSG athletes. Data are presented as means ± standard deviations.

Figure 1 presents the findings from the three-day observational period. Athletes typically woke up between 10:00 and 10:30 a.m. Following eating snacks, they began the morning team training session at 11:00 a.m., which lasted six hours. During the training session, athletes were in a seated position. From 5:00 p.m. to 7:00 p.m., the team held a mandatory 40-min exercise session. Over the three observational days, this session involved group jogging near their training base. Minute-level heart rate analysis revealed that athletes spent an average of 35.6 min per day engaged in MVPA, with an average heart rate of 141 beats per minute. After the exercise session, athletes rested and had dinner at the training base. Between 7:00 p.m. and 11:00 p.m., athletes participated in a nighttime team training session. At 11:00 p.m., the mandatory training concluded, after which athletes often ordered delivery food for a light meal. Starting around midnight, all athletes voluntarily conducted late-night training sessions. Athletes continued training until 3:00 a.m., after which they ended their day and went to bed. On average, each athlete consumed two large cups of coffee and 2.5 bottles of energy drinks daily. They also drank various sugary beverages, such as Coca-Cola, throughout the day. Alcoholic beverages were strictly prohibited during training and match days, as informed by the team coach. Based on Apple Watch metrics, the athletes’ average time spent awake in bed was 45.2 ± 13.0 min, total sleep time was 392.8 ± 13.9 min, and sleep efficiency was 89.7 ± 3.0%.

## 4 Discussion

This study present new evidence from an elite professional esports team demonstrating that RSG athletes often engage in sedentary physical activity and exhibit abnormal sleep patterns during their daily training routines. To combat fatigue from insufficient sleep or maintain high vigilance for gaming, these athletes frequently consume stimulant drinks. These preliminary findings offer valuable insights into the health challenges faced by Chinese professional esports athletes and highlight the need for promoting health behaviors within the rapidly growing industry.

A key criterion distinguishing professional esports athletes from amateur players is the duration of daily practice. For example, RSG athletes dedicate 13 h per day to training, which aligns with the practice routines of South Korean professional esports athletes, who typically train 13.4 h daily (2). In contrast, non-professional players train significantly fewer hours (22, 23). China and South Korea lead the world in professional esports industry development, consistently producing top-ranked athletes in various game titles, such as League of Legends and Valorant. To remain competitive in their typical 3–5 year professional careers, elite esports athletes must commit substantial time to refining individual skills, team strategies, and adapting to rivals’ advancements. It is unsurprising that professional esports athletes from China and South Korea train more than 10 h daily throughout their careers. At RSG, athletes are required to train for 10 h per day, focusing on individual skills and team tactics. This schedule adheres to China’s labor laws. Additionally, RSG athletes voluntarily extend their training by another four hours daily to enhance their game proficiency, contributing to their recent success in qualifying for the PUBG Mobile Global Championship 2024. Thus, training for over 10 h daily is a defining characteristic of professional esports athletes.

As a result, RSG athletes spend the majority of their active time engaged in sedentary behaviors. Previous reports on professional esports athletes have highlighted the prevalence of overuse-related musculoskeletal injuries, including neck pain, lower back pain, and poor spinal posture (7, 8, 24). Although the injury profiles of RSG athletes has not been assessed, prolonged sitting time is associated with lower back pain, particularly when sitting with awkward spinal postures (25). Moreover, the specific type of esports game that RSG athletes compete in adds additional strain on their neck, shoulder, and forearm muscles. Unlike traditional esports games that rely on a computer screen, mouse, and keyboard, PUBG Mobile is a smartphone-based game. RSG athletes must hold a touchscreen smartphone with both hands while playing, which necessitates looking down at the screen for extended periods. Empirical studies have shown that excessive time spent gaming on smartphones can lead to awkward postures, causing muscle fatigue and discomfort, particularly in the posterior neck area (26). Prolonged smartphone use activates the neck extensor muscles (27) and may be linked to cervical disc degeneration, contributing to conditions like cervical spondylosis (28). Given the extremely long daily training hours spent in a seated position — reading and tapping on a touchscreen — RSG athletes are theoretically more prone to chronic musculoskeletal injuries in the neck, lower back, shoulders, thumbs, and forearms. They may also face other common strains, such as vision and auditory impairments. It is possible that team managers recognize the hazards of prolonged sedentary behaviors and have mandated a daily exercise session to mitigate these risks. While the team managers’ efforts to improve the physical health of their athletes should be acknowledged, the overall ratio of physically active time to sedentary time still suggests a disproportionate exposure to occupational health risks.

In addition to prolonged periods of daytime physical inactivity, RSG athletes demonstrated abnormal sleep patterns compared to traditional norms. Similar to South Korean professional esports athletes (2), RSG athletes exhibited delayed sleep schedules, with a typical bedtime of 3:05 a.m. Sleep metrics further revealed that RSG athletes experienced prolonged awake periods, averaging 45.2 min. This extended awake time could be attributed to increased sleep latency resulting from heightened cognitive arousal, potentially caused by exposure to gaming background music (29). Alternatively, it is possible that athletes spent idle time in bed browsing their smartphones, a common habit among Chinese adolescents (30). Regardless of the cause, these delayed sleep patterns compromised overall sleep hygiene. On average, RSG athletes achieved only 6.5 h of total sleep, with a sleep efficiency of just 89.7%. To our knowledge, this is the shortest total sleep time reported in the literature on professional esports athletes. In comparison, South Korean professional esports athletes have been reported to sleep longer — approximately 7 h — though this duration is still insufficient and associated with daytime sleepiness (31).

There are three main health concerns regarding the abnormal sleep patterns of RSG athletes: delayed bedtimes, insufficient sleep duration, and poor sleep quality. First, delayed bedtimes directly contribute to shorter total sleep time, which is an obvious consequence. However, less obvious repercussions may involve other health behaviors. A cross-sectional study on sleep habits among adolescents and young adults found that late bedtimes were associated with lower odds of consuming healthy foods, higher odds of consuming unhealthy foods and beverages, more frequent fast-food consumption, and greater intake of sugar-sweetened beverages (32). This pattern was also evident in RSG athletes. They regularly skipped a quality breakfast, opting instead for calorie-dense snacks before the morning training session. Their reliance on prepared delivery foods, known to be associated with increased all-cause mortality (33), and their nighttime eating habits, which elevate the risk of cardiovascular diseases (34), are concerning, to put it mildly. Additionally, their frequent consumption of sugary drinks is a well-documented contributor to various metabolic disorders.

Second, sleep deprivation can lead to daytime fatigue. A previous study of 50 professional esports athletes competing in the Onmyoji Arena Pro League reported that 46% frequently felt tired (5). Consequently, professional esports athletes often rely on stimulant drinks to sustain vigilance and maintain optimal gaming performance. In this study, RSG athletes consumed, on average, two large cups of coffee and two to three bottles of energy drinks daily. This is the first report in the literature to provide such detailed consumption patterns among professional esports athletes. The situation is further complicated by the fact that energy drink producers are frequent sponsors of esports competitions and individual teams (ZY’s under review manuscript titled “Implied continuity and diversification of corporate sponsorship managerial strategies in the League of Legends Pro League”), exposing athletes to significant commercial influences. There is substantial evidence that short sleep duration is independently associated with both the prevalence and incidence of atrial fibrillation (35). While the link between caffeine-loaded energy drink consumption and atrial fibrillation remains controversial (36, 37), international health communities are increasingly cautious about the cardiovascular risks associated with excessive energy drink use (38). Chronic sleep deprivation combined with the excessive intake of coffee and energy drinks may trigger sudden cardiac events, posing life-threatening occupational risks for professional esports athletes.

Third, habitual short sleep duration may lead to the development of chronic noncommunicable diseases. For instance, Eide and colleagues demonstrated through *in vivo* evidence that a single night of sleep deprivation impaired the clearance of tracer substances from most brain regions, and this impaired cerebral clearance cannot be compensated by subsequent sleep (39). This suggests that professional esports athletes may face an elevated risk of beta-amyloid accumulation during their professional years, which could potentially contribute to their typically short professional careers due to slowed reaction times critical for elite gaming performance. Furthermore, optimal immune system function depends on adequate sleep quality to maintain its various roles (40). Sleep deprivation in both human and animal studies has been associated with up-regulated inflammatory markers (41, 42), potentially leading to chronic low-grade organ damages and disease development. While the esports industry is relatively young, making it premature to definitively claim that a professional esports career increases the risk of chronic noncommunicable diseases, the overarching concern remains valid: poor sleep quality is a significant health risk for this population.

## 5 Practical implications

Taken together, these findings highlight several intervention opportunities to safeguard the well-being of professional esports athletes. Considering the extended hours athletes spend in a seated position, it would be worthwhile for the gaming peripheral industry to explore the development of ergonomic gaming chairs that provide enhanced lumbar and spinal support. Such chairs could ideally incorporate features allowing athletes to alternate between sitting and standing during gameplay. Additionally, implementing structured breaks during training sessions may mitigate the risks associated with overuse injuries. For instance, if teams were to mandate a 10-min break every hour during training, incorporating targeted exercises such as neck and lower back stretches or rope-jumping could benefit athletes’ MVPA. Given recent evidence suggesting that no amount of MVPA can offset the harms of prolonged sedentary behavior (6), all industry stakeholders should be made aware of this risk. Regulations enforcing an upper limit on total work hours for professional esports players may be worth considering. The importance of proper nutrition also warrants attention. Providing basic nutritional education could encourage healthier dietary habits, such as reducing sugar-sweetened beverage consumption and integrating nutrient-rich foods into daily routines. Such changes might also optimize brain function (43), a critical factor for competitive gaming performance. Sleep hygiene is another area that deserves intervention. Modifying indoor environmental parameters, such as optimizing lighting and reducing noise levels (44), may help improve athletes’ sleep quality and recovery. Furthermore, medical preparedness should be standardized across esports organizations. Given the potential risks of sudden cardiac events, it is essential that governing bodies of esports competitions and leagues consider mandating annual certified cardiopulmonary resuscitation training for team workers. Additionally, ensuring the availability of life-saving equipment, such as automated external defibrillators, could be a prerequisite for team registration in official leagues. Addressing these issues could greatly enhance the health and safety of professional esports athletes, ensuring their longevity in the profession and fostering the sustainable growth of this burgeoning industry.

## 6 Limitations and future research

Finally, a few study design-related issues warrant clarification. While it might be tempting to critique the generalizability of this case study’s findings to other Chinese professional esports athletes, readers should recognize that extremely long training hours and short sleep duration are defining features of the professional esports ecosystem in China. Conversations with coaches from RSG and other teams confirm that no athlete in this industry enjoys the luxury of an eight-hour workday. Instead, the expectation is clear: athletes must train to their maximum capacity until in-game actions become instinctive. Those who fail to meet this rigorous standard risk being removed from the roster and payroll. Although there are expected differences in training hours and bedtime routines across teams and individuals, the overarching daily structure for professional esports athletes in China remains consistent: relentless training dominates their schedules. While exercise behaviors, such as the extent of MVPA and energy drink consumption, vary depending on each team’s culture, one conclusion remains evident. Chinese professional esports athletes spend the majority of their active hours seated during training days. Overall, it is our opinion that this case study reflects the typical training environment of Chinese professional esports athletes.

A limitation of this study was the lack of cardiac function measurements, such as atrial fibrillation, which could theoretically be observed in professional esports athletes with chronic sleep deprivation. Currently, only the Apple Watch offers this feature, requiring a costly paired iPhone for proper reporting. However, as other manufacturers introduce similar capabilities in consumer health monitoring devices, future research could gain deeper insights into the effects of poor sleep quality on esports athletes’ cardiac health. The standard adoption of these devices could further enhance the safety and well-being of professional esports athletes.

## 7 Conclusive remarks

The strength of this descriptive case study lies in its measurement of real-world health behaviors from an elite esports team competing at the highest level in China. These firsthand findings reveal critical issues such as prolonged sedentary behavior and severely reduced sleep duration. Additionally, the long-term effects of frequent stimulant drink consumption may pose further health concerns. These insights underscore the need for targeted interventions by team managers and industry regulators. With thoughtful adjustments, it is possible to promote the physical well-being of athletes while ensuring the sustainable development of the esports industry.

## Data Availability

The raw data supporting the conclusions of this article will be made available by the authors, without undue reservation.
